# Influence of driving pressure and tidal volume on survival of unselected patients requiring mechanical ventilation: a prospective longitudinal study in the ICUs of a quaternary academic medical center in Brazil

**DOI:** 10.36416/1806-3756/e20250282

**Published:** 2026-11-09

**Authors:** Daniele Robira, Larissa Padrão Isensee, Marina Lavezzo dos Santos Vilar, Fernando Felix da Silva, Heloisa Francelin, José Otávio Costa Auler, Líria Yuri Yamauchi, Carlos Roberto Ribeiro de Carvalho, Marcelo Park, Carolina Fu

**Affiliations:** 1. Departamento de Fisioterapia, Fonoaudiologia e Terapia Ocupacional, Faculdade de Medicina, Universidade de Sao Paulo, Sao Paulo (SP) Brasil.; 2. Departamento de Cirurgia, Faculdade de Medicina, Universidade de Sao Paulo, Sao Paulo (SP) Brasil.; 3. Departamento de Ciências de Movimento Humano, Universidade Federal de São Paulo, Santos (SP) Brasil.; 4. Divisão de Pneumologia, Faculdade de Medicina, Universidade de Sao Paulo, Sao Paulo (SP) Brasil.; 5. Laboratório de Investigação Médica - Emergências Médicas, Faculdade de Medicina, Universidade de Sao Paulo, Sao Paulo (SP) Brasil.

**Keywords:** Ventilator-induced lung injury, Pressure, Tidal volume, Survival analysis, Critical care, Respiration, artificial

## Abstract

**Objective:**

To evaluate the association of driving pressure (ΔP) and V_T_ with survival in unselected patients on mechanical ventilation (MV) in a quaternary care hospital in Brazil, as well as whether the initial behavior of ΔP influenced the number of ventilator-free days.

**Methods:**

This was a single-center prospective cohort study of hospitalized adults across 13 ICUs of an academic medical center in the city of São Paulo, Brazil. We analyzed the first instance of invasive MV ≥ 24 h. Patients were followed for up to 60 days after ICU admission. We used Cox regression to analyze patient survival in relation to ΔP and V_T_ on the first day of MV. The number of ventilator-free days was compared between patients in whom ΔP decreased on the first three days of MV and those in whom ΔP increased or remained stable.

**Results:**

A total of 231 patients were evaluated. Of those, 57% were male. The mean age was 55 ± 18 years. The mean Simplified Acute Physiology Score III was 61 ± 17. The median Charlson Comorbidity Index was 3 [IQR, 1-4]. Survival analysis showed that neither ΔP (relative risk = 1.04; 95% CI, 0.08-0.99; p = 0.09) nor V_T_ (relative risk = 0.98; 95% CI, 0.90-1.07; p = 0.66) on the first day of MV affected survival. Disease severity (Simplified Acute Physiology Score III; p < 0.005) and comorbidities (Charlson Comorbidity Index; p < 0.005) were crucial factors for mortality.

**Conclusions:**

In our cohort of mechanically ventilated patients with high disease severity and comorbidity burden, neither ΔP nor V_T_ on the first day of MV was associated with survival. No ventilation variable predicted survival, which was primarily determined by disease severity and comorbidities. Changes in ΔP over the first three days were not associated with differences in the number of ventilator-free days.

## INTRODUCTION

Mechanical ventilation (MV) is an essential resource for advanced life support in patients with respiratory failure. Mechanical ventilator-induced lung injury is an adverse effect of MV, and inappropriate management of MV may trigger or aggravate acute lung injury or ARDS.^1^ Protective ventilatory strategies may reduce mortality in ARDS patients^2^ ^-^ ^4^ and benefit patients with normal lungs.^5^

Certain ventilatory parameters are considered to be predictive of mortality, including V_T_, driving pressure (ΔP), plateau pressure (Pplat), and PEEP. In patients with ARDS, ventilation with high V_T_ is associated with poorer outcomes, supporting the use of a V_T_ of < 6 mL/kg.^3^ ^,^ ^6^ Pressure has also been shown to have a strong association with mortality.^2^ ^,^ ^7^ Specifically, a ΔP below 15 cmH_2_O^8^ has been used as a protective strategy in patients with ARDS.^7^ ^,^ ^8^ In normal lungs, strategies with low V_T_ and ΔP have been associated with a lower incidence of pulmonary complications^9^ ^,^ ^10^; however, threshold values and their association with mortality are unclear.^11^ ^-^ ^15^

Poorer Simplified Acute Physiology Score III (SAPS III) outcomes have been reported in South America and Central America in comparison with other regions, including Australasia, Western Europe, Eastern Europe, Northern Europe, Southern Europe, Mediterranean countries, and North America.^16^ Although MV is well studied, lung protective parameters are more clearly defined in patients with ARDS, whereas evidence in patients with normal lungs remains inconsistent regarding direct associations of ΔP and V_T_ with mortality. To improve outcomes and reduce harm, it is essential to identify ventilation strategies tailored to specific populations. New data can therefore complement existing evidence and help optimize care for critically ill patients. 

The objective of the present study was to evaluate the influence that ΔP and V_T_ adjusted for the predicted body weight on the first day of MV had on the survival of unselected patients on MV in a quaternary academic medical center in the city of São Paulo, Brazil. We also assessed whether the initial behavior of ΔP influenced the number of ventilator-free days. Our hypothesis was that elevated ΔP and V_T_ normalized to normal lung volume are associated with reduced survival in patients with and without pulmonary disease, including ARDS. 

## METHODS

### Study design and settings

This was a prospective observational cohort study. The study was conducted across 13 ICUs: 10 at the *Instituto Central* and 3 at the *Hospital do Coração*, both within the *Hospital das Clínicas* medical center of the University of São Paulo Medical School. Data were collected from March of 2019 to March of 2020. Patients were followed from the first instance of invasive MV for more than 24 h up to 60 days after ICU admission. We analyzed the association of ΔP and predicted body weight-adjusted V_T_ on the first day of MV with survival. 

The present study was approved by the Research Ethics Committee of the institution (CAPPesq; Protocol no. 2.89.805). 

### Participants

We included patients > 18 years of age on invasive MV for more than 24 h for the first time after admission to the ICU. We excluded cases of patients with suspected bronchopleural fistula, in which it was impossible to measure lung mechanics, as well as cases of unlikely Pplat values, with Pplat ≤ PEEP. 

### Data collection

Previously trained researchers collected data using a manual of procedures. Patient data collection began upon ICU admission at initiation of MV. Data were included if the patient remained on MV for at least 24 h. Data collection was interrupted by the COVID-19 pandemic, and we therefore used a convenience sample. 

### Variables

Descriptive characteristics were recorded on admission, including identification data, demographics, severity scales,^16^ comorbidities,^17^ hospitalization, and clinical information. A diagnosis of ARDS was defined as an ARDS entry on the medical record of the patient. 

Outcome variables were obtained by analyzing the dates. Survival was obtained by analyzing the date of hospital discharge or death, as well as patient status, i.e., alive or dead within 60 days of admission to the ICU. The data were retrieved from electronic medical records or by contacting patients or their family members via telephone. The number of MV-free days was obtained in accordance with Yehya et al.,^18^ with censoring at 28 days; for analysis, we considered the number of days without MV and alive ≤ 28 days. The follow-up period was = 60 days from the ICU admission date. For patients who were discharged and for whom follow-up data on vital status within 60 days of admission to the ICU were unavailable, survival was assumed. 

MV variables were collected daily during the morning shift from study inclusion until MV discontinuation by direct bedside observation of the ventilator. The study primary analysis was based on ΔP and predicted body weight-adjusted V_T_ as measured on the first day of MV, within a 24-36-h window. MV variables included the following: ventilation mode; PEEP; FiO_2_; V_T_; total respiratory rate; inspiratory time; and peak pressure (Ppeak). For Pplat, we considered an inspiratory pause pressure > 2 s on volume-controlled mode; on pressure control ventilation, we considered the Ppeak value, with inspiratory time > 1 s, without signs of auto-PEEP.^19^ ^,^ ^20^ On pressure support mode, we assumed that the Pplat value equal to the inspiratory pause pressure value, like pressure muscle index maneuver^21^ we made the measurement at the daily collection. For the PaO_2_/FiO_2_ ratio, we checked FiO_2_ at the time of test collection. The primary study variables were ΔP, which was calculated by subtracting Pplat from PEEP,^7^ and V_T_, which was divided by the predicted body weight. The predicted body weight was calculated as follows: 50 + 0.91(height − 152.4) for males and 45.5 + 0.91(height − 152.4) for females.^22^

### Statistical analysis

Categorical variables were presented as mean ± standard deviation or median and interquartile range, and absolute numbers and proportions were used for qualitative data. The Shapiro-Wilk test was used in order to verify data normality. For the analysis, the sample was divided into two groups on the basis of mortality: survivors and nonsurvivors. For group comparison, the Student’s t-test or the Mann-Whitney test was applied for quantitative variables, and the chi-square test was used for categorical variables. 

To illustrate the survival of the sample, we used the Kaplan-Meier method.^23^ A multivariate Cox regression model was used in order to answer the primary question of the study. The choice of variables for inclusion in the model was based on information from the univariate analysis and a directed acyclic graph.^24^ The dependent variables were ΔP and V_T_, and the independent variables were SAPS III, the Charlson Comorbidity Index, and the cause of intubation. Two survival analyses were conducted: one for ΔP and one for V_T_. For the representation, we used the variables in categories without proportionality between them and presented the distribution of each category. Thus, we verified the joint action of possible risk factors for survival in relation to the main variables of the study. To improve the sensitivity of the Cox regression model, we used the recursive partitioning method.^25^ ^-^ ^27^ We presented the results through the representation of the classification tree. 

To assess whether the early trajectory of ΔP influenced the number of ventilator-free days, we compared patients whose ΔP remained the same or increased from day 1 to day 3 with those whose ΔP decreased during the same period. Mortality was analyzed by group, and the number of ventilator-free days was compared by using the log-rank test. 

All analyses were performed with the IBM SPSS Statistics software package, version 21.0 (IBM Corporation, Armonk, NY, USA) and Python. All tests were two-tailed, with values of p < 0.05 being considered significant. 

## RESULTS

Of the 253 patients who met the inclusion criteria during the study period, 22 were excluded, the final sample therefore consisting of 231 patients. Figure 1 shows the study flowchart. 

**Figure 1:**
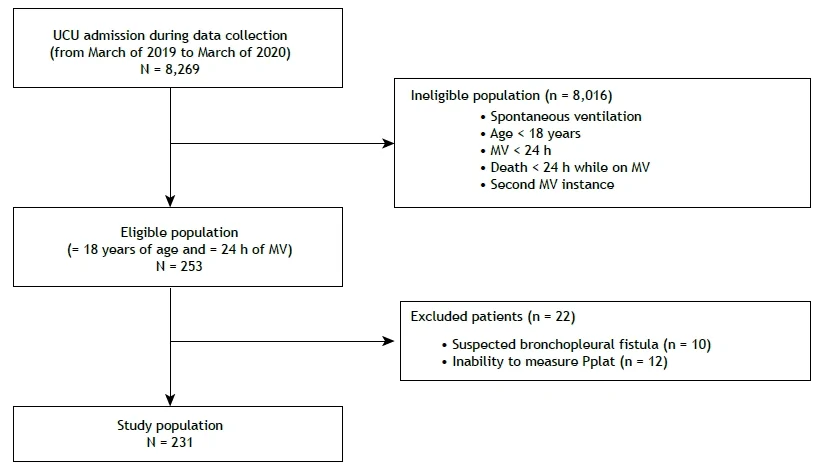
Study flowchart. MV: mechanical ventilation; and Pplat: plateau pressure.

The study sample was predominantly male, with a mean age of 55 years. The population showed a high severity profile on admission, with a mean SAPS III of 61 and a median Charlson Comorbidity Index of 3. Most ICU admissions were for clinical reasons, and the leading causes of intubation were neurological events and perioperative procedures. The overall mortality was 54%. When survivors and nonsurvivors were compared, significant differences were found in age, SAPS III, and the Charlson Comorbidity Index. These and other clinical characteristics are detailed in Table 1. 

**Table 1:** Clinical characteristics and outcomes of patients on mechanical ventilation at ICU admission, including the overall population and a comparison between survivors and nonsurvivors.^a^

Variable	Total N = 231	Survivors n = 107	Nonsurvivors n = 124	p
Clinical characterization				
Age, years*	55 ± 18	50 ± 17	59 ± 18	< 0.001
Male sex^†^	132 (57)	59 (55)	73 (59)	0.568
SAPS III*	61 ± 17	55 ± 15	66 ± 17	< 0.001
Charlson Comorbidity Index^‡^	3 [1-4]	2 [0-3]	3 [2-5]	< 0.001
BMI, kg/m^2^	25 [22-29]	25 [22-29]	25 [23-28]	0.939
ICU admission cause^†^				0.579
Clinical	126 (55)	56 (52)	70 (57)	
Elective postoperative	41 (18)	22 (21)	19 (75)	
Urgency postoperative	64 (28)	29 (27)	35 (28)	
Initial diagnosis				
Neurological	66 (29)	36 (34)	30 (24)	
Respiratory	50 (22)	22 (21)	28 (23)	
Cardiovascular	51 (22)	28 (26)	23 (19)	
Gastrointestinal	34 (15)	10 (9)	19 (15)	
Other	30 (13)	11 (10)	19 (15)	
Heart failure^†^	23 (10)	9 (8)	14 (11)	0.466
Lung disease^†^	26 (11)	8 (8)	18 (15)	0.870
COPD	12 (5)	5 (5)	7 (6)	0.729
Cystic fibrosis	2 (1)	-	2 (2)	1.000
Interstitial disease	3 (1)	-	3 (2)	0.250
Pulmonary hypertension	1 (0.5)	-	1 (1)	0.500
Asthma	7 (3)	2 (2)	5 (4)	0.454
ARDS^†^	17 (7)	11 (10)	6 (5)	0.114
Sepsis^†^	6 (5)	1 (2)	5 (7)	0.410
Type of ICU^†^				0.057
Medical ICU	86 (37)	42 (39)	44 (35)	
Surgical ICU	67 (29)	27 (25)	40 (32)	
Trauma ICU	44 (19)	20 (19)	24 (19)	
Cardiovascular ICU	34 (15)	18 (17)	16 (14)	
Intubation cause^†^				0.256
Neurological changes	78 (34)	31 (29)	47 (38)	
Respiratory failure	64 (28)	28 (26)	36 (29)	
Cardiopulmonary arrest	10 (4)	6 (6)	4 (3)	
Procedures	78 (34)	42 (39)	36 (29)	
Clinical outcomes				
Sedation use^†^	180 (78)	76 (71)	104 (84)	
Number of days on sedation^‡^	2 [1-5]	1 [0-5]	2 [1-5]	0.067
Neuromuscular blockade use^†^	27 (11)	8 (7)	21 (16)	
Number of days on neuromuscular blockade*	0.4 ± 1.5	0.3 ± 1.1	0.6 ± 1.7	0.115
Vasoactive drugs use^†^	175 (76)	64 (59)	112 (90)	
Number of days on vasoactive drug^‡^	2 [1-4]	1 [0-4]	2 [0-4]	< 0.001
Reintubation^†^	42 (18)	15 (14)	27 (21)	0.128
Length of stay, days^‡^	20 [10-34]	26 [16-41]	14 [8-26]	< 0.001
Length of ICU stay, days^‡^	10 [6-18]	13 [8-20]	8 [4-15.5]	< 0.001
Duration of MV, days^‡^	4.7 [2.13-8.4]	4 [2-8]	5 [2-9]	0.268
Number of ventilator-free days^‡^	11 [0-23]	23 [18-26]	0 [0-0]	< 0.001
Mortality	124 (54)	-		
Death after hospital discharge	12 (5)	-	12 (10)	

SAPS III: Simplified Acute Physiology Score III. ^a^Values expressed as mean ± SD, n (%), or median [IQR]. *Student’s t-test. ^†^Chi-square test. ^‡^Mann-Whitney test.

No significant differences were observed between survivors and nonsurvivors regarding MV parameters on the first day. ΔP, V_T_, and Pplat were within lung protective targets. Respiratory system compliance was slightly reduced across the cohort. Full ventilatory data are presented in Table 2. 

**Table 2:** Characterization of the study population based on mechanical ventilation parameters on the first day of ventilation, including the overall population and a comparison between survivors and nonsurvivors.

Variable	Total N = 231	Survivors n = 107	Nonsurvivors n = 124	p
PaO_2_/FiO_2_ ratio*	257 [162-370]	297 [170-505]	240 [148-345]	0.113
Crs, cmH_2_O*	36 [28-48]	35 [28-47]	37 [2949]	0.771
pH*	7.36 [7.28-41]	7.36 [7.30-7.41]	7.34 [7.25-7.41]	0.288
PaCO_2_, mmHg*	40 [34.5- 44.8]	41.4 [35.6 45.2]	38.6 [32.9-44.0]	0.051
ΔP, cmH_2_O*	12 [10- 15]	12 [10-15]	12 [10-15]	0.484
V_T_, mL/kg*	7.6 [6.6-8.8]	7.5 [6.5-8.7]	7.6 [6.6-8.8]	0.904
Pplat, cmH_2_O*	19 [16-22]	19 [17-22]	19 [16-22]	0.739
PEEP, cmH_2_O*	6 [6- 8]	6 [6-8]	6 [6-8]	0.300
Respiratory rate, bpm*	20 [16- 24]	19 [16-24]	20 [18-24]	0.169
Ventilator mode				0.517
Spontaneous mode^†^	51 (22)	27 (25)	24 (19)	
Pressure support mode^†^	163 (71)	73 (68)	90 (73)	
Volume-controlled mode^†^	17 (7)	7 (7)	10 (8)	
Advanced strategies for hypoxemia^†^	10 (4)	5 (5)	5 (5)	> 0.999
Alveolar recruitment	2 (20)	1 (3)	1 (2)	> 0.999
PEEP table	0			
Prone position	5 (50)	3 (9)	2 (3)	0.333
Nitric oxide	1 (10)	0	1 (2)	> 0.999
ECMO	1 (10)	0	1 (2)	> 0.999

Crs: respiratory system compliance; ΔP: driving pressure; Pplat: plateau pressure; and ECMO: extracorporeal membrane oxygenation. ^a^Values expressed as median [IQR] or n (%). *Mann-Whitney test. †Chi-square test.

To understand whether the primary variables, i.e., ΔP and predicted body weight-adjusted V_T_, interfered with the survival of our sample, we performed a multivariate Cox regression analysis. The behavior of ΔP and V_T_ was evaluated separately, including the SAPS III and Charlson Comorbidity Index variables in the models, both of which had been the significant variables in the univariate analysis, and the variable cause of intubation, which had not been significant in the univariate analysis. 

Table 3 presents the Cox regression results for both models. ΔP was not associated with survival (hazard ratio [HR] = 1.04; 95% CI, 0.08-0.99; p = 0.09), whereas SAPS III (HR = 1.03; 95% CI, 1.01-1.04; p < 0.005) and the Charlson Comorbidity Index (HR = 1.16; 95% CI, 0.21-1.08; p < 0.005) were. Similarly, predicted body weight-adjusted V_T_ on the first day of MV was not associated with survival (HR = 0.98; 95% CI, 0.90-1.07; p = 0.66), whereas SAPS III (HR = 1.03; 95% CI, 1.02-1.04; p < 0.005) and the Charlson Comorbidity Index (HR = 1.16; 95% CI, 1.08-1.23; p < 0.005) remained associated with survival. 

**Table 3:** Multivariate Cox regression models: association of survival with driving pressure and tidal volume adjusted for predicted body weight on the first day of mechanical ventilation.

Variable	HR	95% CI	p
Model for the driving pressure			
ΔP	1.04	0.08-0.99	0.09
SAPS III	1.03	1.01-1.04	< 0.005
Charlson Comorbidity Index	1.16	0.21-1.08	< 0.005
Orotracheal intubation causes			
Neurological	0.16	0.02-1.02	0.070
Respiratory	0.08	−0.46 to 0.01	0.020
Cardiopulmonary arrest	0.06	−0.60 to 0.01	0.010
Procedures	0.06	−0.70 to 0.01	0.010
Model for the tidal volume			
V_T_, mL/kg	0.98	0.90-1.07	0.660
SAPS III	1.03	1.02-1.04	< 0.005
Charlson Comorbidity Index	1.16	1.08-1.23	< 0.005
Orotracheal intubation causes			
Neurological	0.16	0.02-1.24	0.080
Respiratory	0.09	0.01-0.68	0.020
Cardiopulmonary arrest	0.06	0.01-0.57	0.010
Procedures	0.07	0.01-0.52	0.010

ΔP: driving pressure; SAPS III: Simplified Acute Physiology Score III; V_T_: tidal volume adjusted for predicted body weight; and HR: hazard ratio.

For the graphic representation of the survival curve, we applied the variables categorically, dividing them into four groups without proportionality between them. We used the threshold values based on the literature for ΔP^7^ ^,^ ^8^ ^,^ ^28^ and V_T_.^2^ ^,^ ^11^ ^,^ ^29^ Thus, we were able to observe the survival curves separately. The intersection of the curves demonstrated that ΔP and V_T_ were not associated with the survival of the study sample. Figure 2 shows the sample survival curves as a function of ΔP and V_T_ adjusted for predicted body weight. 

**Figure 2:**
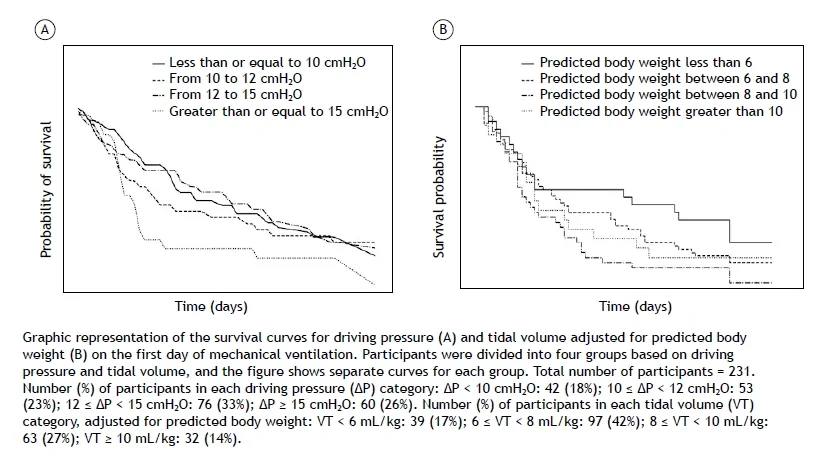
Survival as a function of driving pressure (in A) and tidal volume adjusted for predicted body weight (in B) on the first day of mechanical ventilation. Note: Participants (N = 231) were divided into four groups on the basis of driving pressure (ΔP) and predicted body weight-adjusted tidal volume (V_T_). The figure shows separate curves for each group. The number (%) of participants in each ΔP category was as follows: ΔP < 10 cmH_2_O, 42 (18%); 10 ≤ ΔP < 12 cmH_2_O, 53 (23%); 12 ≤ ΔP < 15 cmH_2_O, 76 (33%); and ΔP ≥ 15 cmH_2_O, 60 (26%). The number (%) of participants in each V_T_ category was as follows: V_T_ < 6 mL/kg, 39 (17%); 6 ≤ V_T_ < 8 mL/kg, 97 (42%); 8 ≤ V_T_ < 10 mL/kg, 63 (27%); and V_T_ ≥ 10 mL/kg, 32 (14%).

A sensitivity analysis was performed to enhance the results. We used the recursive partitioning model, with the variables SAPS III, Charlson Comorbidity Index, ΔP, and V_T_ represented in Figure 3. The model demonstrated that a very high SAPS III (i.e., > 60) was associated with a high chance of dying (70%); when SAPS III was < than 60, a high Charlson Comorbidity Index (i.e., ≥ 4) became associated with a high chance of dying. When SAPS III and the Charlson Comorbidity Index were low, the likelihood of mortality was reduced. At no time during partitioning was ΔP or V_T_ identified, not even in situations of low severity and few comorbidities, indicating that, for our sample, the variables did not modulate the chance of dying. 

**Figure 3:**
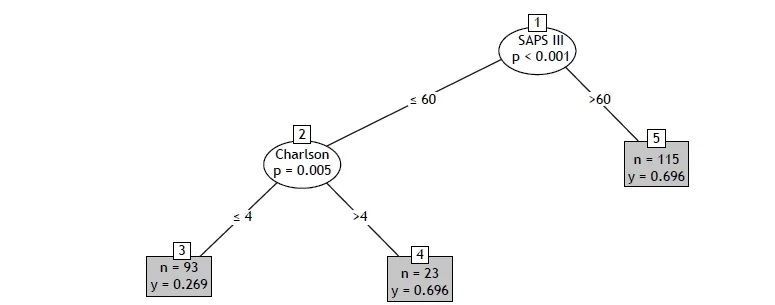
Recursive partitioning for driving pressure and tidal volume adjusted for predicted body weight on the first day of mechanical ventilation. SAPS III: Simplified Acute Physiology Score III; and Charlson: Charlson Comorbidity Index.

Finally, we analyzed the first three days of MV in the study sample with regard to ΔP values. Patients were divided into two groups: group A included those in whom ΔP remained the same or increased from day 1 to day 3; and group B included those in whom ΔP decreased during the same period, reflecting patients who showed improvement in lung mechanics versus those who did not improve or worsened. For this analysis, we excluded patients who died between 24 h and 48 h of MV-three in group A and one in group B. We assessed mortality by group and compared the number of ventilator-free days using the log-rank test. No significant difference was observed in the number of ventilator-free days between the two groups. 

## DISCUSSION

The present study analyzed the survival of a cohort of unselected patients on invasive MV for more than 24 h at a quaternary care hospital in Brazil in relation to two ventilation variables, namely, ΔP and V_T_. Our results showed that neither ΔP nor V_T_ influenced survival. These findings align with those reported in larger single-center and multicenter cohort studies conducted primarily in high-income countries in Europe and North America.^10^ ^,^ ^13^ ^,^ ^30^ However, in our cohort, disease severity and comorbidity burden emerged as key determinants of mortality, highlighting the particularities of critically ill populations in middle-income countries and reinforcing the need for context-specific evidence. 

The mortality rate in our study population was 54%. According to one study, hospital mortality is associated with several factors at admission,^31^ including acute disease severity, patient age, comorbidities, and type of surgery. Our analyses showed that mortality in the study population was mainly associated with the risk factors identified at ICU admission, namely, disease severity, comorbidities, and patient age. 

Mortality in our population was high: a SAPS III of 61 predicts approximately 50% hospital mortality,^16^ slightly lower than observed in the present study. In a prospective cohort study conducted in Brazil,^32^ mortality was found to be 50.9% with lower SAPS III, likely reflecting the exclusion of patients with ARDS in the first 48 h of MV. In a retrospective study conducted in a tertiary hospital in Brazil, mortality was found to be lower, although the study included patients with and without MV; MV is linked to pulmonary complications and worse prognosis, particularly in cases of prolonged ventilation, sepsis, and advanced age,^29^ ^,^ ^33^ ^-^ ^35^ all of which are factors that were observed in our cohort. 

In general, studies on MV identify disease severity and comorbidities as significant risk factors for mortality. Sensitivity analysis reinforces this finding and shows that if a patient is severely ill and has comorbidities, MV is not a risk factor for mortality, although MV may still influence mortality in patients with lung impairment.^7^

In patients with ARDS, lung compliance is related to lung injury and survival.^28^ Reduced compliance directly reflects on ΔP. However, it is not possible to extend this relationship to patients without ARDS. In our study sample, lung compliance values were below normal, which may have influenced clinical decisions regarding ventilation management, particularly the choice of protective MV strategies. Even so, no association with survival was found for any of the variables, including respiratory system compliance and ΔP. Schmidt et al.^13^ found no association between ΔP and mortality in a cohort study involving a larger sample. They argue that this association in patients without ARDS should be expected because lung compliance is not a risk factor in such patients. Our findings confirm the results of that study.^13^ They show that although ΔP is unlikely to be associated with mortality, it might be associated with other outcomes. 

In a secondary analysis of an international multicenter prospective study,^10^ no association was found between ΔP and hospital mortality, with a low V_T_ (7.9 (6.8-9.1) mL/kg) leading to lower ΔP (12.0 (9.0-15.0) cmH_2_O) being cited as a possible consequence. This could also have occurred in our study in situations with lower values. Another vital point to consider is that although ΔP is a care variable in patients with ARDS, we still lack strong evidence to consider ΔP a protective ventilation strategy in patients without ARDS.^30^

Some studies have found an association between ΔP and mortality, such as that by Sahetya et al.,^14^ in which ΔP was higher than it was in the aforementioned studies, with a mean value of 14.6 ± 6.0 cmH_2_O. The study sample was characterized by older patients, with most admissions being due to respiratory causes, 31% being due to sepsis, and 30% being due to pneumonia, with high severity indicators and comorbidities. These are risk factors for developing ARDS,^36^ and the recommendation in such cases is for less permissive ventilation.^37^ For this profile of patients, protective ventilation may be necessary. 

In a randomized clinical trial comparing low V_T_ (< 6 mL/kg) with intermediate V_T_ (6-10 mL/kg),^12^ no impact on mortality was found, although low V_T_ was associated with respiratory acidosis. Reduced V_T_ can decrease alveolar ventilation, causing hypercapnia and pH changes. Although compensation by increasing respiratory rate is possible, it is less physiological than adjusting V_T_ and may lead to patient-ventilator asynchrony, greater sedative use, and higher delirium risk. 

Our sample was characterized by a median V_T_ of 7.6 mL/kg, which has been described as intermediate V_T_.^29^ Neither pH nor PaCO_2_ was associated with mortality, with values remaining at normal or well-tolerated levels. Maintaining V_T_ between 6 mL/kg and 8 mL/kg is closer to physiological ventilation^38^ and safer if we consider the positive effects reported in the literature, including reduced lung injury, length of stay, and exposure to acidosis and asynchronies, all of which are factors that may have an impact on the prognosis of patients with severe disease and comorbidities. 

In our study, ventilatory parameters were determined by the clinical care team and the ventilation provided can be considered predominantly protective, given that its variability remained within established safety limits. This finding may be explained by the study setting, i.e., academic medical centers where clinical practices are typically guided by current evidence-based literature. The routine implementation of ventilation strategies with parameters below critical thresholds was likely promoted by this institutional culture. 

With regard to strengths and limitations, our study was a single-center study conducted at quaternary care hospitals, which receive patients previously screened for severity by the health care network. This might have selected a sample of patients with more risk factors for mortality. To deal with the confounding bias inherent to observational studies, we used a causal directed acyclic graph for inferences. 

The median ΔP in our cohort was 12 (10-15) cmH_2_O, i.e., within the protective range, which may have attenuated the adverse effects of MV. The inclusion of an unselected ICU population likely reflects routine practice and enhances external validity, although potential effects on subgroups with greater sensitivity to respiratory mechanics may have been diluted. Future studies should assess whether these findings persist in specific populations, such as patients with greater disease severity or a higher prevalence of ARDS. 

Because we analyzed MV only on the first day, our findings may not represent the entire ventilation period. Because of the observational design, Pplat was assessed during spontaneous ventilatory modes without sedation, although it is ideally measured on volume-controlled ventilation with neuromuscular blockade. Based on prior evidence^21^ and trained assessment, we used an indirect approach, acknowledging potential imprecision but supporting its feasibility.^28^ Previous studies have shown that inspiratory occlusion during pressure support ventilation yields Pplat and ΔP values comparable to those yielded by volume-controlled ventilation,^21^ supporting the validity of this strategy in real-world conditions. 

In conclusion, in a cohort of patients receiving invasive MV and presenting with high disease severity and comorbidity scores, and in whom the applied ventilation settings were predominantly protective, no association was observed between survival and either ΔP or V_T_ on the first day of ventilation. Disease severity and comorbidities were the main determinants of survival. Additionally, no MV variable was associated with survival in this patient population. There was no significant difference in the number of ventilator-free days between patients in whom ΔP decreased over the first three days of MV and those in whom it remained stable or increased. 
